# Supplementary material for: Sensitive period for rescuing parvalbumin interneurons connectivity and social behavior deficits caused by TSC1 loss
Source: Nat Commun. 2021 Jun 16;12:3653. doi: 10.1038/s41467-021-23939-7 (PMC8209106; doi:10.1038/s41467-021-23939-7)
Supplement: Supplementary file 1 — Supplementary Information [file 41467_2021_23939_MOESM1_ESM.pdf]

Sensitive period for rescuing parvalbumin interneurons connectivity and social behavior deficits caused by *TSC1* loss

Amegandjin[#], Choudhury[#] et al.

Supplementary Table

Gene	Name	Direction	Sequences
TSC1	F4536	Fwd	5'-AGGAGGCCTCTTCTGCTACC-3'
TSC1	R4830	Rev	5'-CAGCTCCGACCATGAAGTG-3'
TSC1	R6548	Rev	5'-TGGGTCCTGACCTATCTCCTA-3'
Nkx2.1Cre	F1	Fwd	5'-AAGGCGGACTCGGTCCACTCCG-3'
Nkx2.1Cre	F2	Fwd	5'-TCCTCCAGGGGACTCAAGATG-3'
Nkx2.1Cre	R1	Rev	5'-TCGGATCCGCCGCATAACCAG-3'
PVCre	148-454-Fwd	Fwd	5'-CAGCCTCTGTTCCACATACACTCC-3'
PVCre	149-o106m-Fwd	Fwd	5'- GCTCAGAGCCTCCATTCCCT-3'
PVCre	150-o103m-Rev	Rev	5'-TCACTCGAGAGTACCAAGCAGGCAGGAGATATC-3'
RCE	RCE-Rosa1	Fwd	5'-CCCAAAGTCGCTCTGAGTTGTTATC-3'
RCE	RCE-Rosa2	Fwd	5'-GAAGGAGCGGGAGAAATGGATATG-3'
RCE	RCE-Cag3	Rev	5'-CCAGGCGGGC CATTTACCGTAAG-3'

Supplementary Table 1. List of primers used for mouse genotyping.

Supplementary Figures

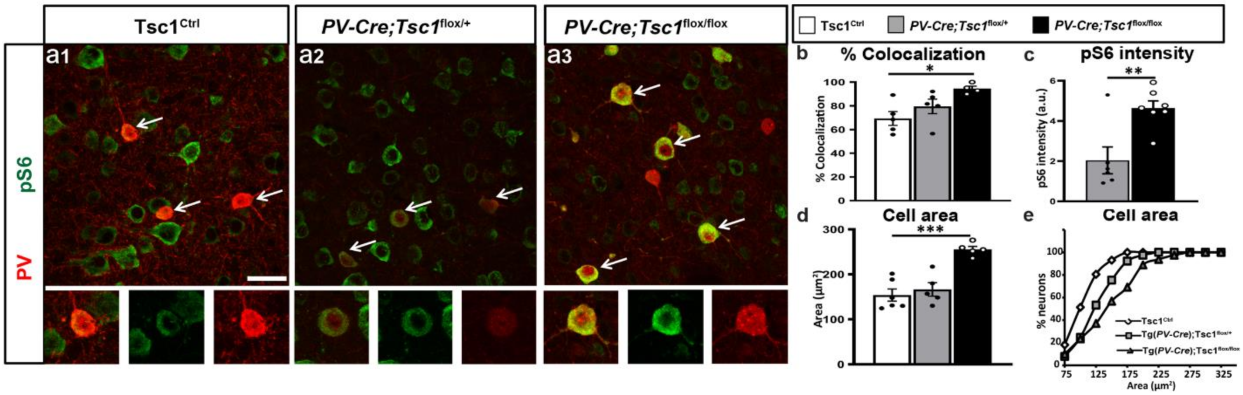

Supplementary Figure 1. Cortical PV cells show increased mTOR activity and somatic hypertrophy in *PV-Cre;Tsc1$^{flox/flox}$* mice. **a**, Coronal sections of somatosensory cortex immunostained for PV (red) and pS6 (green) in *Tsc1*^{Ctrl} (**a1**), *PV-Cre*;*Tsc1$^{flox/+}$* (**a2**) and *PV-Cre*;*Tsc1$^{flox/flox}$* mice (**a3**) at P45. Lower panels show individual PV cells. **b**, *PV-Cre*;*Tsc1$^{flox/flox}$* mice show increased percentage of colocalization of pS6 in PV cells (One-way ANOVA, *p=0.0254; Holm-Sidak's multiple comparisons: *Tsc1*^{Ctrl} **vs** *PV-Cre*;*Tsc1$^{flox/+}$* p=0.1929; *Tsc1*^{Ctrl} **vs** *PV-Cre*;*Tsc1$^{flox/flox}$* *p=0.0239). Number of mice: *Tsc1*^{Ctrl} n = 5, *PV-Cre*;*Tsc1$^{flox/+}$* n= 5, *PV-Cre*;*Tsc1$^{flox/flox}$* n= 4. **c**, Quantification of pS6 expression intensity in PV cells normalized to wild-type controls show two-fold increase in *PV-Cre;Tsc1$^{flox/flox}$* mice (Welch's t-test **p=0.0093). Number of mice: *PV-Cre;Tsc1$^{flox/+}$* n= 6, *PV-Cre*;*Tsc1$^{flox/flox}$* n= 7. **d, e,** PV cells show somatic hypertrophy in homozygous mutant mice. **d** (One-way ANOVA, ***p=0.0001; Dunnett's multiple comparisons: *Tsc1*^{Ctrl} **vs** *PV-Cre*;*Tsc1$^{flox/+}$* p=0.7005; *Tsc1*^{Ctrl} vs *PV-Cre*; *Tsc1$^{flox/flox}$* ***p=0.0001). Number of mice: *Tsc1*^{Ctrl} n = 6, *PV-Cre*;*Tsc1$^{flox/+}$* n= 5, *PV-Cre*;*Tsc1$^{flox/flox}$* n= 5. **e**, Cumulative distribution (K-S test, *p<0.001) at P45. Scale bar, 20 µm. Data represent mean ± SEM.

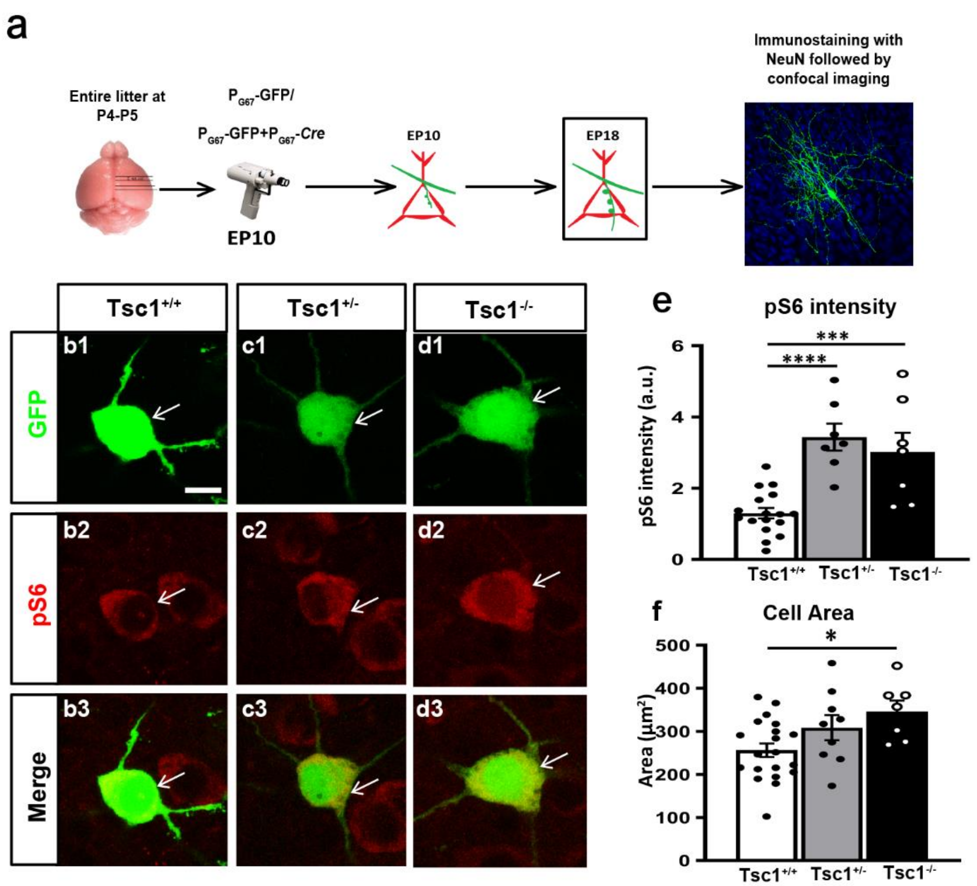

Supplementary Figure 2. *Tsc1* knockout in single PV cells leads to increase in mTOR activity and somatic hypertrophy. a, Schematics of experimental procedure. **b-d,** PV cells from cortical organotypic cultures transfected with P_{G67} (**b**, *Tsc1*$^{+/+}$ control cells) or P_{G67}-Cre (**c**, *Tsc1*$^{+/-}$ and **d**, *Tsc1*$^{-/-}$) at EP10 (P5+5 days *in vitro=Equivalent Postnatal day 10*) and immunostained for pS6 (red) at EP18 (P5+13 days *in vitro=Equivalent Postnatal day 18*). **e**, Somatic pS6 intensity is increased in both *Tsc1*$^{+/-}$ and *Tsc1*$^{-/-}$ PV cells compared to *Tsc1*$^{+/+}$ PV cells (One-way ANOVA, ****p<0.0001; Holm-Sidak's multiple comparisons: *Tsc1*$^{+/+}$ **vs** *Tsc1*$^{+/-}$ ****p<0.0001; *Tsc1*$^{+/+}$ **vs** *Tsc1*$^{-/-}$ ***p=0.0003). Number of cells: *Tsc1*$^{+/+}$ n = 17, *Tsc1*$^{+/-}$ n=7, *Tsc1*$^{-/-}$ n=7. **f**, *Tsc1*$^{-/-}$ cells have increased soma area (One-way ANOVA, *p=0.0213; Holm-Sidak's multiple comparisons: *Tsc1*$^{+/+}$ **vs** *Tsc1*$^{+/-}$ p=0.1675; *Tsc1*$^{+/+}$ **vs** *Tsc1*$^{-/-}$ *p=0.0277). Number of transfected cells: *Tsc1*$^{+/+}$ n=20, *Tsc1*$^{+/-}$ n=9, *Tsc1*$^{-/-}$ n=7. Scale bar, 10µm. Data represent mean ± SEM.

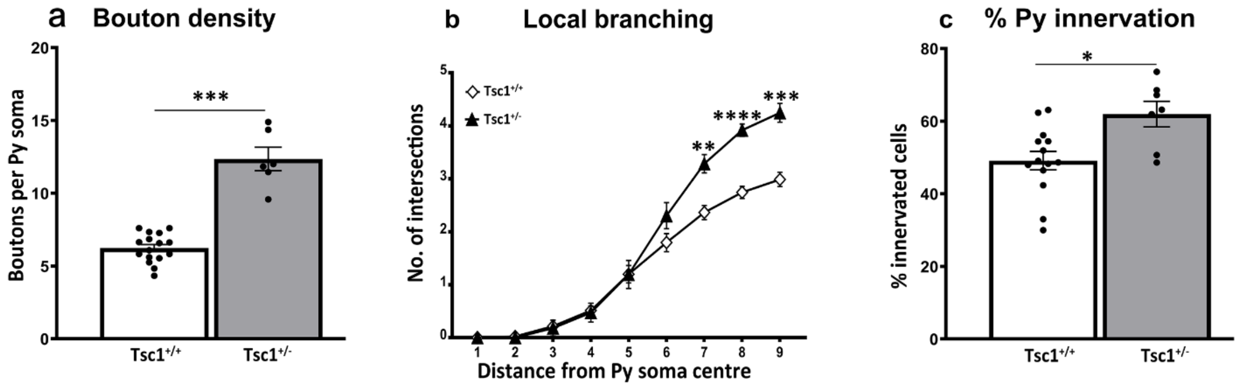

Supplementary Figure 3. *Tsc1* haploinsufficiency in single PV cells causes a premature increase in axon terminal branching and bouton density. a, PV cells lacking one allele of *Tsc1* show increase in bouton density (Welch's t-test, ***p=0.0004), number of PV cells: n=16 for *Tsc1*$^{+/+}$, n=6 for *Tsc1*$^{+/-}$ and local branching (**b**) (Welch's t-test, **p=0.0014 (radius7), ****p<0.0001 (radius8), ***p=0.0001 (radius9)), number of PV cells: n=15 for *Tsc1*$^{+/+}$, n=6 for *Tsc1*$^{+/-}$. **c**, Percentage of innervated cells (Welch's t-test, *p=0.0114). Number of PV cells: n=14 *Tsc1*$^{+/+}$, n=7 *Tsc1*$^{+/-}$. Data represent mean ± SEM.

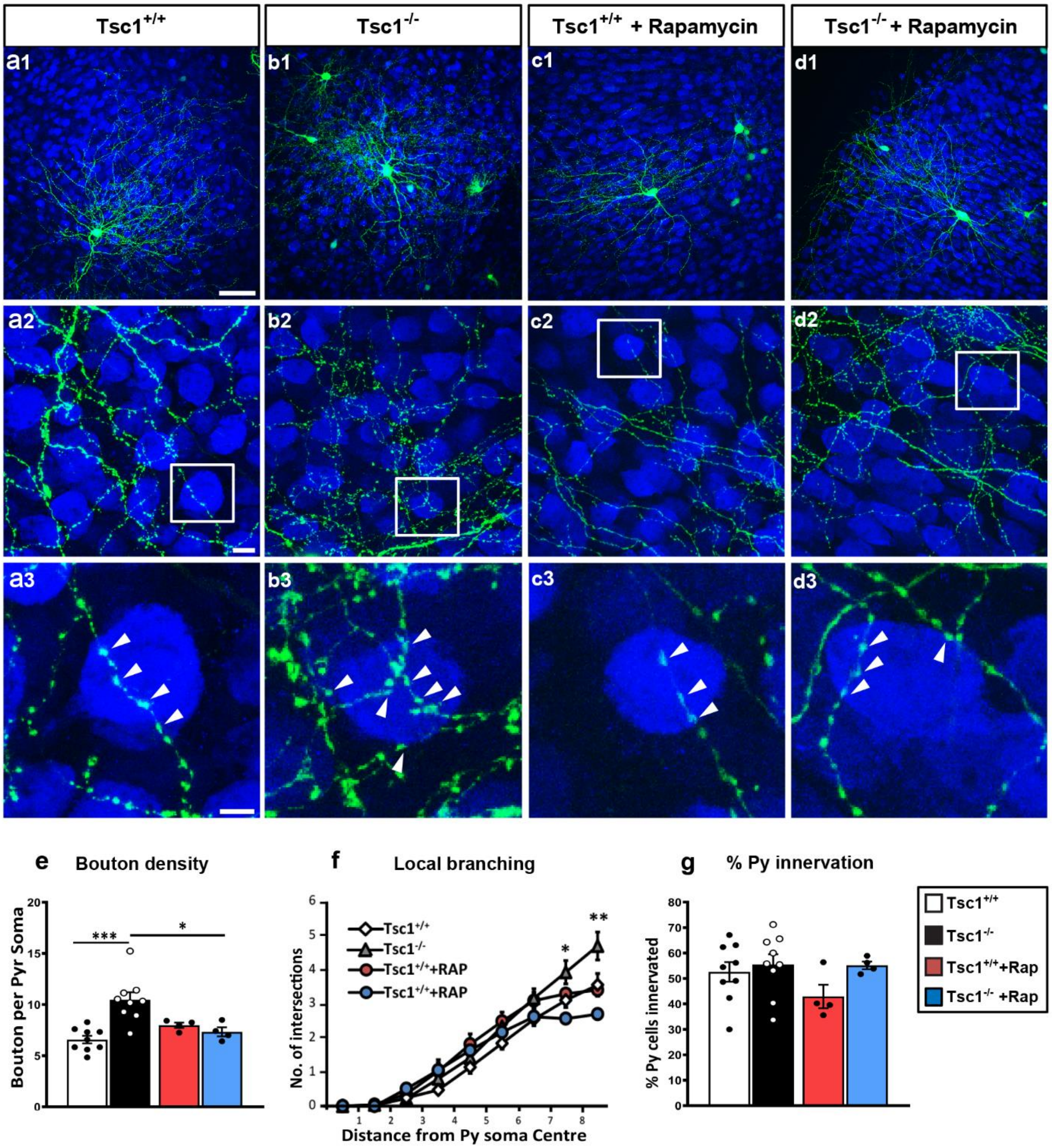

Supplementary Figure 4. Premature increase in perisomatic innervation by *Tsc1*$^{-/-}$ PV cells is mTORC1 dependent. a, b, *Tsc1*$^{-/-}$ PV cell (green) shows more complex terminal axonal branching (**a2, b2)** and increased bouton density at EP18 (**a3, b3,** arrowheads) compared to control, age-matched PV cells. **c, d,** Rapamycin treatment from EP12-18 does not affect bouton density and local branching of *Tsc1*$^{+/+}$ PV cells (**c**), while it normalizes perisomatic innervations formed by *Tsc1*$^{-/-}$ PV cells (**d**). *Tsc1*$^{-/-}$ PV cells show increased bouton density (**e**) (Two-way ANOVA, $F_{treatment}$ (1, 22)=1.763 p=0.1979, $F_{genotype}$ (1, 22)=6.165 p=0.0211, $F_{genotype*treatment}$ (1,

22)=12.05 p=0.0022; *p=0.0129, ***p=0.0001; Tukey's multiple comparisons test; number of PV cells: n=9 *Tsc1*$^{+/+}$, n=9 *Tsc1*$^{-/-}$, n=4 *Tsc1*$^{+/+}$ + Rapamycin, n=4 *Tsc1*$^{-/-}$ + Rapamycin) and local branching (**f**) (Two-way ANOVA, F_{radius} (1.532, 16.85)=42.94 p<0.0001, $F_{Treatement}$(3, 11)=0.9657 p=0.4433, $F_{genotype}$(11, 88)=22.27 p<0.0001, $F_{radius*treatment}$ (24, 88)=1.521 p=0.0820; *p<0.05, **p<0.001; Bonferroni's multiple comparisons test; number of PV cells: n=4 *Tsc1*$^{+/+}$, n=3 *Tsc1*$^{-/-}$, n=4 *Tsc1*$^{+/+}$ + Rapamycin, n=4 *Tsc1*$^{-/-}$ + Rapamycin;) compared to the other groups. **g**, Percentage of innervation (Two-way ANOVA, $F_{treatment}$ (1, 22)=1.207 p=0.2837, $F_{genotype}$ (1, 22)=2.782 p=0.1095, $F_{genotype*treatment}$ (1, 22)=1.049 p=0.3168; number of PV cells: n=9 *Tsc1*$^{+/+}$, n=9 *Tsc1*$^{-/-}$, n=4 *Tsc1*$^{+/+}$ + Rapamycin, n=4 *Tsc1*$^{-/-}$ + Rapamycin). Scale bars: **a1-d1**, 100 µm; **a2-d2** and **a3-d3**, 5 µm. Data represent mean ± SEM.

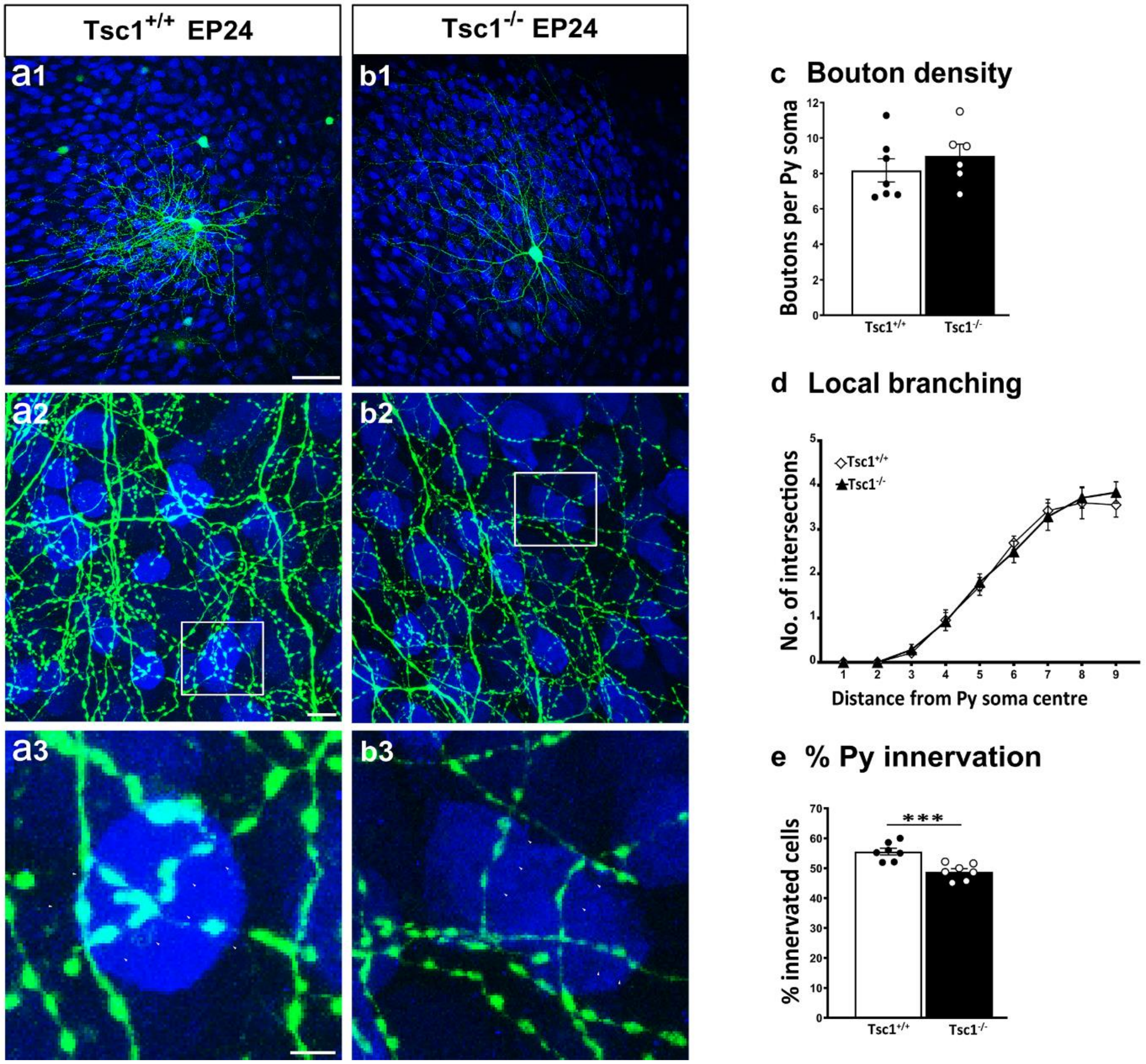

Supplementary Figure 5. PV *Tsc1*$^{-/-}$ cell innervations at EP24 are morphologically indistinguishable from age-matched controls. a1, *Tsc1*$^{+/+}$ and **b1**, *Tsc1*$^{-/-}$ PV cells show similar axonal branching (**a2, b2**) and perisomatic bouton density (**a3, b3,** arrowheads). **c**, Bouton density (Welch's t test, p=0.3920; Number of PV cells: n=7 *Tsc1*$^{+/+}$, n=6 *Tsc*$^{-/-}$). **d,** local branching is not significantly different between the two groups (Number of PV cells: n=5 *Tsc1*$^{+/+}$, n=6 *Tsc*$^{-/-}$). **e**, percentage of innervation is significantly reduced in the *Tsc1*$^{-/-}$ PV cells (Welch's t test, ***p=0.0009; Number of PV cells: n=7 *Tsc1*$^{+/+}$, n=7 *Tsc*$^{-/-}$). Scale bars: **a1-b1**, 50 µm; **a2-b2,** 10 µm, **a3-b3**, 5 µm. Data represent mean ± SEM.

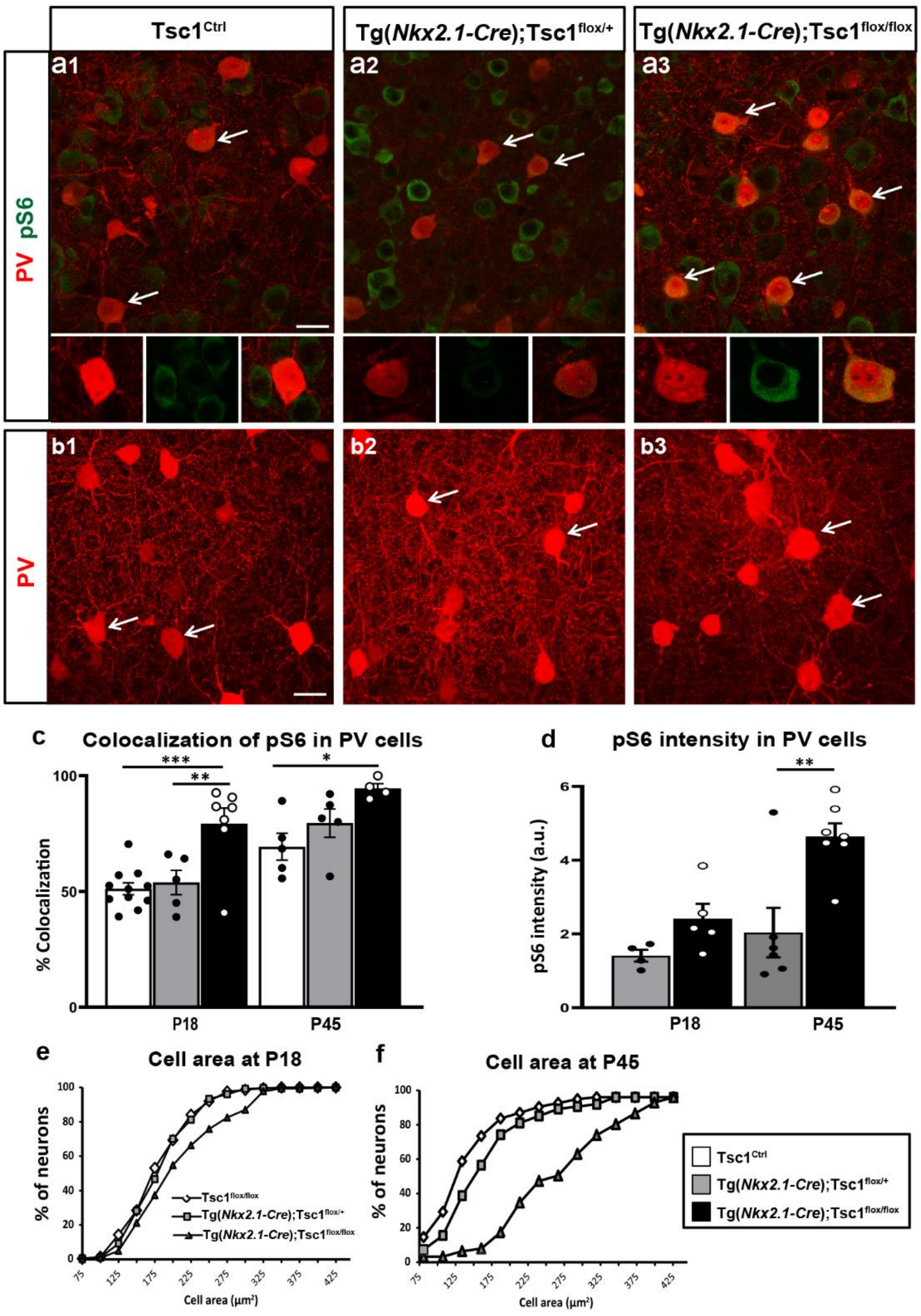

Supplementary Figure 6. Cortical PV cells from Tg(*Nkx2.1-Cre*);*Tsc1*$^{flox/flox}$ mice show increased mTOR activity and somatic hypertrophy. **a**, Coronal sections of somatosensory cortex immunostained for PV (red) and pS6 (green) (**a**) or PV only (**b**) in *Tsc1*^{Ctrl} (**a1, b1**), Tg(*Nkx2.1-Cre*);*Tsc1*$^{flox/+}$ (**a2, b2**) and Tg(*Nkx2.1-Cre*);*Tsc1*$^{flox/flox}$ mice (**a3, b3**) at P18. Lower panels show higher magnification of individual PV cells. **c**, In Tg(*Nkx2.1-Cre*);*Tsc1*$^{flox/flox}$ mice, more PV cells co-localize with pS6 as compared to Tg(*Nkx2.1-Cre*);*Tsc1*$^{flox/+}$ and wild-type mice

at P18 (One-way ANOVA, ***p=0.0005; Tukey's multiple comparison test: *Tsc1*^{Ctrl} vs Tg*(Nkx2.1-Cre);Tsc1*$^{flox/+}$ p=0.9154; *Tsc1*^{Ctrl} **vs** Tg(*Nkx2.1-Cre); Tsc1*$^{flox/flox}$ ***p=0.0005; Tg*(Nkx2.1-Cre);Tsc1*$^{flox/+}$ **vs** *Tg(Nkx2.1-Cre);Tsc1*$^{flox/flox}$ **p=0.0072) and at P45 (One-way ANOVA, *p=0.0254; Tukey's multiple comparison test: *Tsc1*^{Ctrl} vs *Tg(Nkx2.1-Cre);Tsc1*$^{flox/+}$ p=0.3805; *Tsc1*^{Ctrl} vs *Tg(Nkx2.1-Cre);Tsc1*$^{flox/flox}$ *p=0.0202; *Tg(Nkx2.1-Cre);Tsc1*$^{flox/+}$ *Tsc1*^{Ctrl} **vs** Tg*(Nkx2.1-Cre);Tsc1*$^{flox/flox}$ ***p=0.1789). Number of mice at P18 *Tsc1*^{Ctrl} n=11, Tg(*Nkx2.1-Cre); Tsc1*$^{flox/+}$ n=5, *Tg(Nkx2.1-Cre);Tsc1*$^{flox/flox}$ n=7; number of mice at P45, *Tsc1*^{Ctrl} n=5, Tg*(Nkx2.1-Cre);Tsc1*$^{flox/+}$ n=5, *Tg(Nkx2.1-Cre);Tsc1*$^{flox/flox}$ n=4. **d,** pS6 expression intensity in PV cells normalized to wild-type controls at P18 (Welch's t-test p=0.06; number of mice at P18, *Tg(Nkx2.1-Cre);Tsc1*$^{flox/+}$ n=4, *Tg(Nkx2.1-Cre);Tsc1*$^{flox/flox}$ n=5) and at P45 (Welch's t-test **p=0.0093; number of mice at P45, *Tg(Nkx2.1-Cre);Tsc1*$^{flox/+}$ n=6, Tg(*Nkx2.1-Cre*);*Tsc1*$^{flox/flox}$ n= 7. **e, f,** Quantification of PV cell area shows somatic hypertrophy in Tg(*Nkx2.1-Cre*);*Tsc1*$^{flox/flox}$ mice at both P18 and P45 (P18: K-S test, *p<0.01; P45: K-S test, *p<0.001), and in Tg(*Nkx2.1-Cre*); *Tsc1*$^{flox/+}$ mice at P45 (P18: K-S test, *p<0.05), n=11 *Tsc1*^{Ctrl} mice, n=5 Tg(*Nkx2.1-Cre*); *Tsc1*$^{flox/+}$ mice, n=7 Tg(*Nkx2.1-Cre*);*Tsc1*$^{flox/flox}$ mice at P18, n=6 mice for all genotypes at P45. Scale bar, 20 μm. Data represent mean ± SEM.

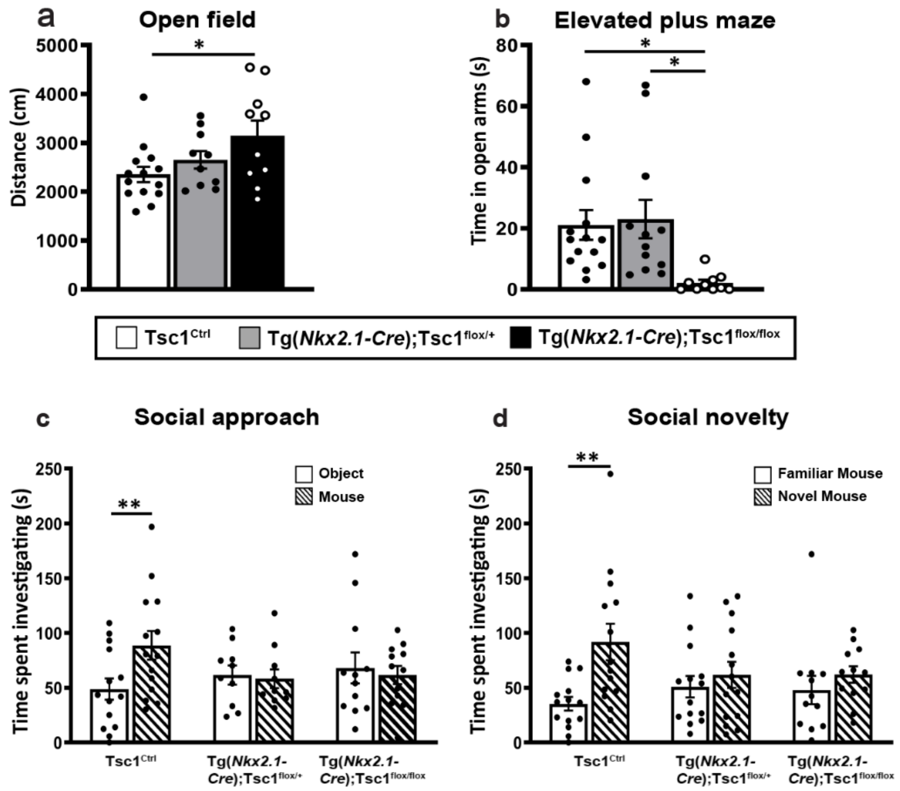

Supplementary Figure 7. *Tsc1* knockout in MGE derived neurons leads to social behavioral deficits in young adult mice. a, Open field test: Quantification of distance travelled during exploratory activity in an open field arena at P33 shows increased exploratory drive in Tg*(Nkx2.1-Cre);Tsc1*$^{flox/flox}$ mice (One-way ANOVA, *p<0.0407; Holm-Sidak's multiple comparisons: *Tsc1*^{Ctrl} **vs** Tg*(Nkx2.1-Cre);Tsc1*$^{flox/+}$ p=0.3246; *Tsc1*^{Ctrl} **vs** Tg*(Nkx2.1-Cre);Tsc1*$^{flox/flox}$ *p=0.0241). Number of mice: *Tsc1*^{Ctrl} n=14, Tg*(Nkx2.1-Cre);Tsc1*$^{flox/+}$ n=10, Tg*(Nkx2.1-Cre); Tsc1*$^{flox/flox}$ n=10. **b**, Elevated plus maze: Quantification of time spent in the open arms of elevated plus maze arena at P35 shows increased anxiety like behavior in Tg*(Nkx2.1-Cre);Tsc1*$^{flox/flox}$ mice (One-way ANOVA, *p=0.0137; Tukey's multiple comparisons test: *Tsc1*^{Ctrl} **vs** Tg*(Nkx2.1-Cre);Tsc1*$^{flx/+}$ p=0.7755; *Tsc1*^{Ctrl} **vs** Tg*(Nkx2.1-Cre);Tsc1*$^{flox/flox}$ *p=0.0230). Number of mice: *Tsc1*^{Ctrl} n=14, Tg*(Nkx2.1-Cre);Tsc1*$^{flox/+}$ n=12, Tg*(Nkx2.1-Cre);Tsc1*$^{flox/flox}$ n=10. **c**, In the 3 chambers, both Tg*(Nkx2.1-Cre);Tsc1*$^{flox/+}$ and Tg*(Nkx2.1-Cre);Tsc1*$^{flox/flox}$ mice do not show preference for the mouse **vs** the object (Two-way ANOVA, $F_{genotype}$ (2, 33)=0.2257 p=0.7992, F_{time} (1, 33)=1.842 p=0.1840, $F_{genotype*time}$ (2, 33)=4.460 p=0.0193; **p=0.0055, Sidak's multiple comparisons test). Number of mice: *Tsc1*^{Ctrl} n=14, Tg*(Nkx2.1-Cre);Tsc1*$^{flox/+}$ n=10, Tg*(Nkx2.1-Cre);Tsc1*$^{flox/flox}$ n=12. **d**, Unlike *Tsc1*^{Ctrl} mice, both Tg*(Nkx2.1-Cre);Tsc1*$^{flox/+}$ and Tg*(Nkx2.1-Cre);Tsc1*$^{flox/flox}$ mice failed to show preference for social novelty (Two-way ANOVA, $F_{genotype}$ (2, 37)=0.2907 p=0.7494, F_{time} (1, 37)=9.499 p=0.0039, $F_{genotype*time}$ (2, 37)=2.877 p=0.0690; **p=0.0016, Sidak's multiple comparisons test). Number of mice: *Tsc1*^{Ctrl} n=14, Tg*(Nkx2.1-Cre);Tsc1*$^{flox/+}$ n=14, Tg*(Nkx2.1-Cre);Tsc1*$^{flox/flox}$ n=12. Data represent mean ± SEM.

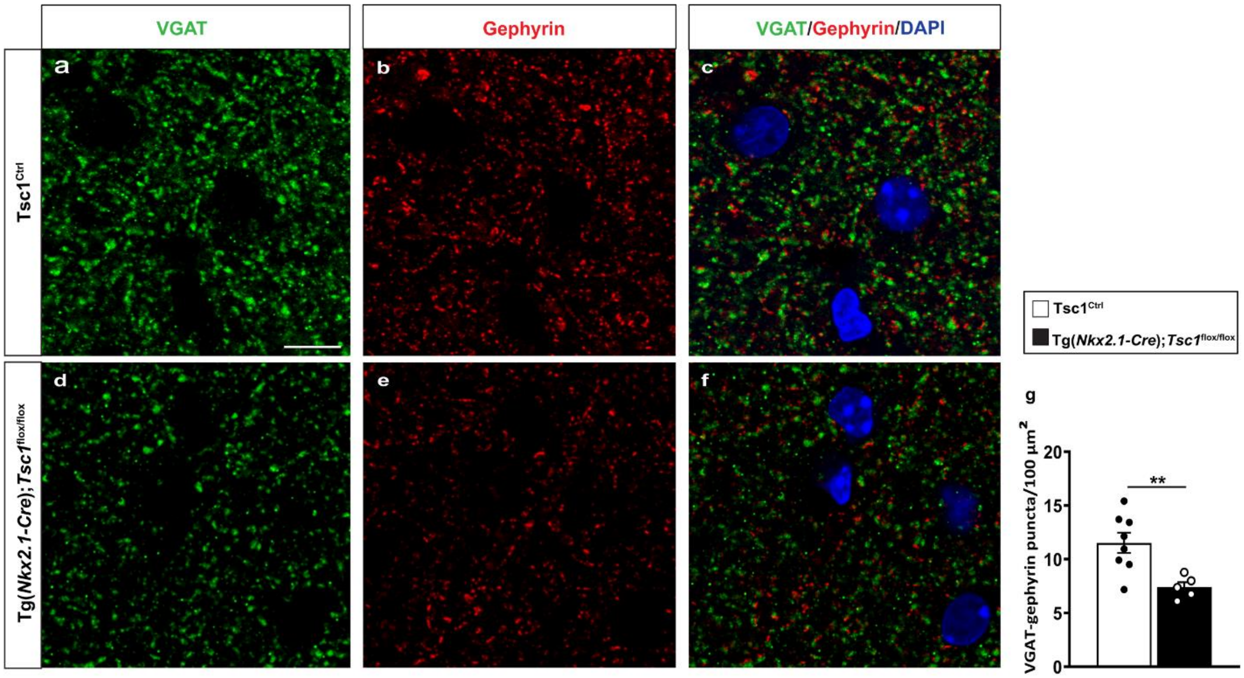

Supplementary Figure 8. ***Tsc1*** **knockout in MGE-derived neurons leads to a significant reduction of GABAergic synapses in the olfactory bulb of adult mice. (a-f)** Horizontal sections of adult mouse olfactory bulb stained for VGAT (green); gephyrin (red) and DAPI (blue). (**g**) Quantification of VGAT/Gephyrin shows a decrease of colocalized puncta (Welch's t test, **p=0.003). Number of mice: *Tsc1*^{Ctrl} n = 8, Tg(*Nkx2.1*-*Cre*);*Tsc1*$^{flox/flox}$ n= 5. Scale bar: 10µm. Data represent mean ± SEM.

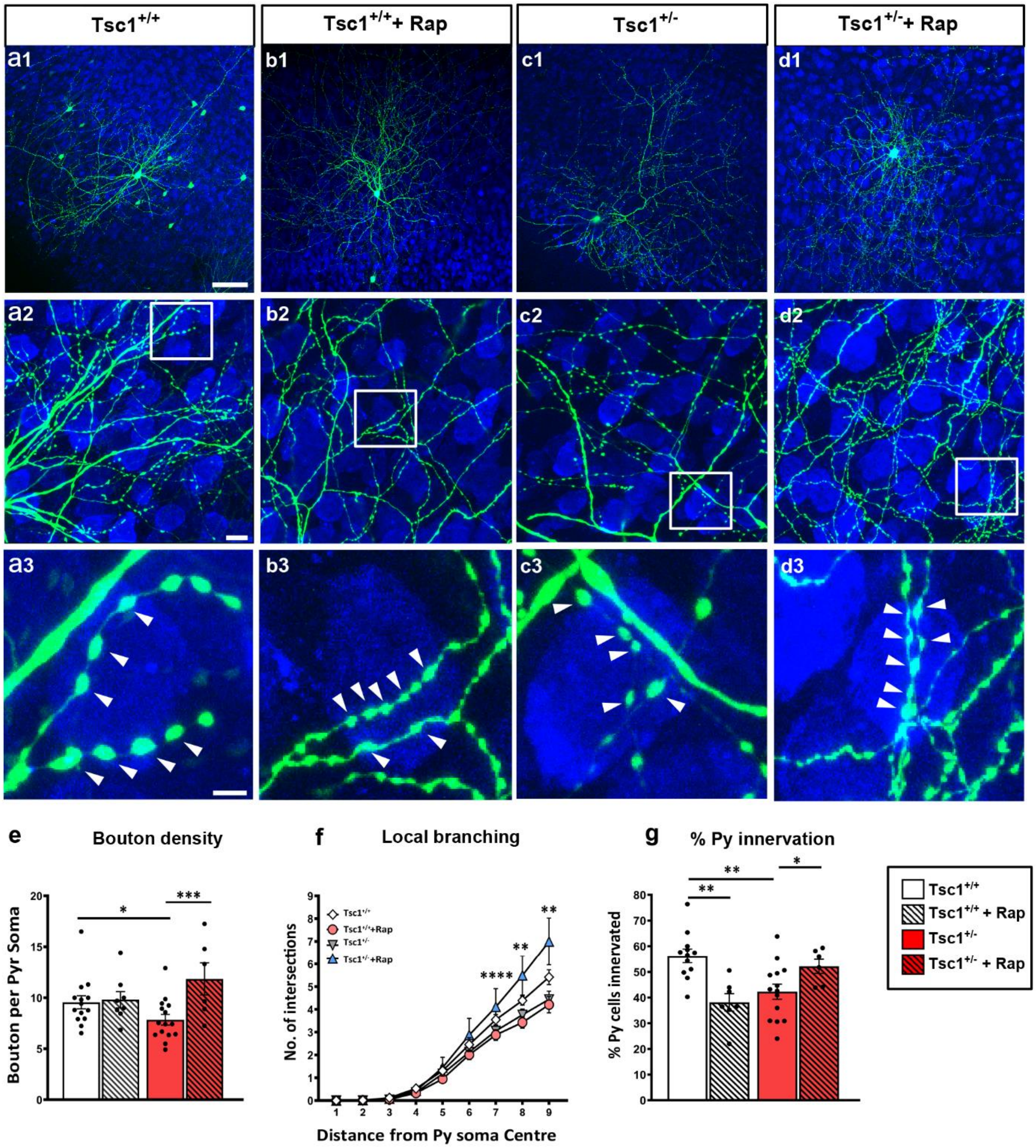

Supplementary Figure 9. Short term Rapamycin treatment rescues loss of perisomatic innervation in Tg*(Nkx2.1-Cre);Tsc1*$^{flox/+}$ mice at EP34. a, b, PV cell (green) among NeuN immunostained neurons (blue) in cortical organotypic cultures from *Tsc1*^{Ctrl} mouse at EP34 where **b** is treated with Rapamycin. **c, d,** PV cells from Tg*(Nkx2.1-Cre);Tsc1*$^{flox/+}$ where **d** is treated with Rapamycin. **e**, Loss of bouton density in PV cells from Tg*(Nkx2.1-Cre);Tsc1*$^{flox/+}$ mice is reversed by Rapamycin treatment (Two-way ANOVA, $F_{\text{treatment}}$ (1, 39)=0.04043 p=0.8417, F_{genotype} (1, 39)=6.755 p=0.0131, $F_{\text{genotype*treatment}}$ (1, 39)=5.252 p=0.0274; *p<0.05, **p<0.001; Bonferroni's multiple comparisons test); PV cells: n=14 *Tsc1*^{Ctrl}, n=14 Tg(*Nkx2.1-Cre*);*Tsc1*$^{flox/+}$, n=8 *Tsc1*^{Ctrl}

+ Rapamycin, n=6 Tg(*Nkx2.1-Cre*); *Tsc1*$^{flox/+}$+ Rapamycin. Similarly, loss of local branching in PV cells from Tg(*Nkx2.1-Cre*);*Tsc1*$^{flox/+}$mice is reversed by Rapamycin treatment (**f**) (Two-way ANOVA, F_{radius} (1.991, 77.65)=308 p<0.0001, $F_{Treatement}$(3, 39)=4.296 p=0.0103, $F_{genotype}$(39, 312)=4.579 p<0.0001, $F_{radius*treatment}$ (24, 312)=3.118 p<0.0001, **p<0.001, ***p<0.001; Bonferroni's multiple comparisons test); PV cells: n=14 *Tsc1*^{Ctrl}, n=15 Tg(*Nkx2.1-Cre*);*Tsc1*$^{flox/+}$, n=8 *Tsc1*^{Ctrl} + Rapamycin, n=6 Tg(*Nkx2.1-Cre*);*Tsc1*$^{flox/+}$+ Rapamycin. **g**, Rapamycin treatment reverses the loss of percentage of innervation in mutant PV cells, while it leads to significant loss of innervation in PV cells from *Tsc1*^{Ctrl} mice (Two-way ANOVA, $F_{genotype}$ (1, 35)=0.0002 p=0.9883, $F_{treatment}$ (1, 35)=1.583 p=0.2167, $F_{genotype*treatment}$ (1, 35)=18.49 p=0.0001, *p<0.05, **p<0.001; Bonferroni's multiple comparisons tes). Arrowheads indicate boutons. PV cells: n=12 *Tsc1*^{Ctrl}, n=14 Tg(*Nkx2.1-Cre*);*Tsc1*$^{flox/+}$, n=7 *Tsc1*^{Ctrl} + Rapamycin, n=6 Tg(*Nkx2.1-Cre*);*Tsc1*$^{flox/+}$ + Rapamycin. Scale bars: **a1-d1**, 10 µm; **a2-d2** and **a3-d3**, 5 µm. Data represent mean ± SEM.

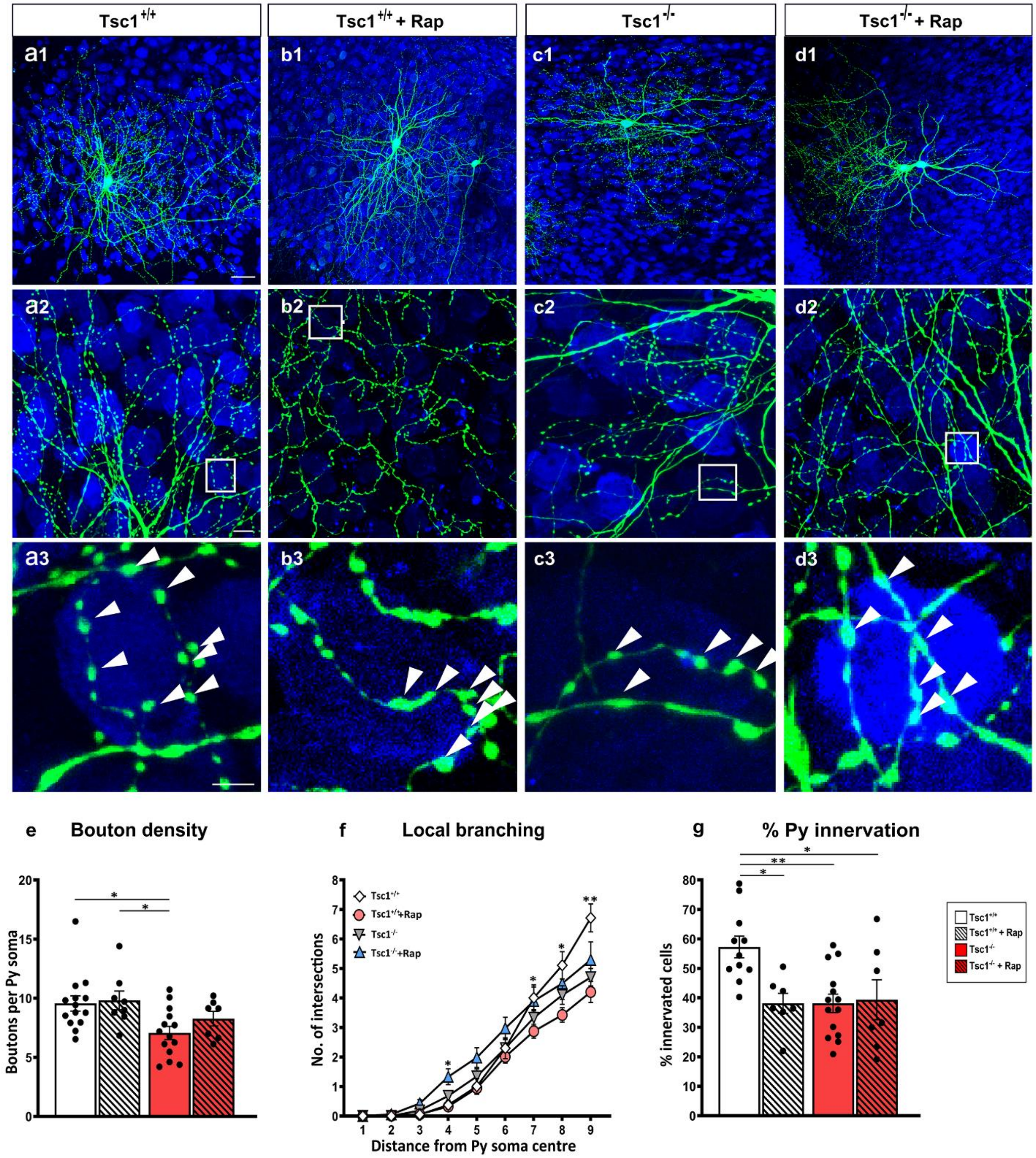

Supplementary Figure 10. Short term Rapamycin treatment does not rescue loss of perisomatic innervations in Tg*(Nkx2.1-Cre);Tsc1*$^{flox/flox}$ mice at EP34. a, b, PV cell (green) among NeuN immunostained neurons (blue) in cortical organotypic cultures from a *Tsc1*^{Ctrl} mouse at EP34 where **b** is treated with Rapamycin. **c, d,** PV cells from Tg(*Nkx2.1-Cre*);*Tsc1*$^{flox/flox}$ where **d** is treated with Rapamycin. **e**, **f**, Loss of bouton density (**e**) (Two-way ANOVA, $F_{treatment}$ (1, 39)=1.100 p=0.3007, $F_{genotype}$ (1, 39)=8.473 p=0.0059, $F_{genotype*treatment}$ (1, 39)=0.4700 p=0.4970;

*p<0.05; Bonferroni's multiple comparisons test; PV cells: n = 14 *Tsc1*^{Ctrl}, n= 14 Tg(*Nkx2.1-Cre*); *Tsc1*$^{flox/flox}$, n= 8 *Tsc1*^{Ctrl} + Rapamycin, n= 7 Tg(*Nkx2.1-Cre*);*Tsc1*$^{flox/flox}$ + Rapamycin) and terminal branching (**f**) (Two-way ANOVA, F_{radius} (8, 304)=388.7 p<0.0001, $F_{Treatement}$(3, 38)=2.238 p=0.0995, $F_{genotype}$(38, 304)=8.573 p<0.0001, $F_{radius*treatment}$ (24, 304)=4.930 p<0.0001, Bonferroni's multiple comparisons test *p<0.05, **p<0.001; PV cells: n = 13 *Tsc1*^{Ctrl}, n= 14 Tg(*Nkx2.1-Cre*);*Tsc1*$^{flox/flox}$, n= 8 *Tsc1*^{Ctrl} + Rapamycin, n= 7 Tg(*Nkx2.1-Cre*);*Tsc1*$^{flox/flox}$ + Rapamycin) in PV cells from Tg(*Nkx2.1-Cre*);*Tsc1*$^{flox/flox}$ mice are only partially reversed by Rapamycin treatment. **g**, Loss of percentage of innervation in PV cells from Tg(*Nkx2.1-Cre*); *Tsc1*$^{flox/flox}$ is not reversed by Rapamycin treatment. Moreover, Rapamycin treatment in *Tsc1*^{Ctrl} mice causes significant loss of innervation (Two-way ANOVA, $F_{treatment}$ (1, 35)=4.422 p=0.0427, $F_{genotype}$ (1, 35)=4.424 p=0.00427, $F_{genotype*treatment}$ (1, 35)=5.720 p=0.0223; Bonferroni's multiple comparisons test, *p<0.05, **p<0.001; PV cells: n=11 *Tsc1*^{Ctrl}, n=14 Tg(*Nkx2.1-Cre*);*Tsc1*$^{flox/flox}$, n=7 *Tsc1*^{Ctrl} + Rapamycin, n=7 Tg(*Nkx2.1-Cre*);*Tsc1*$^{flox/flox}$ + Rapamycin). Arrowheads indicate boutons. Scale bars: **a1-d2**, 10 µm; and **a3-d3**, 5 µm. Data represent mean ± SEM.

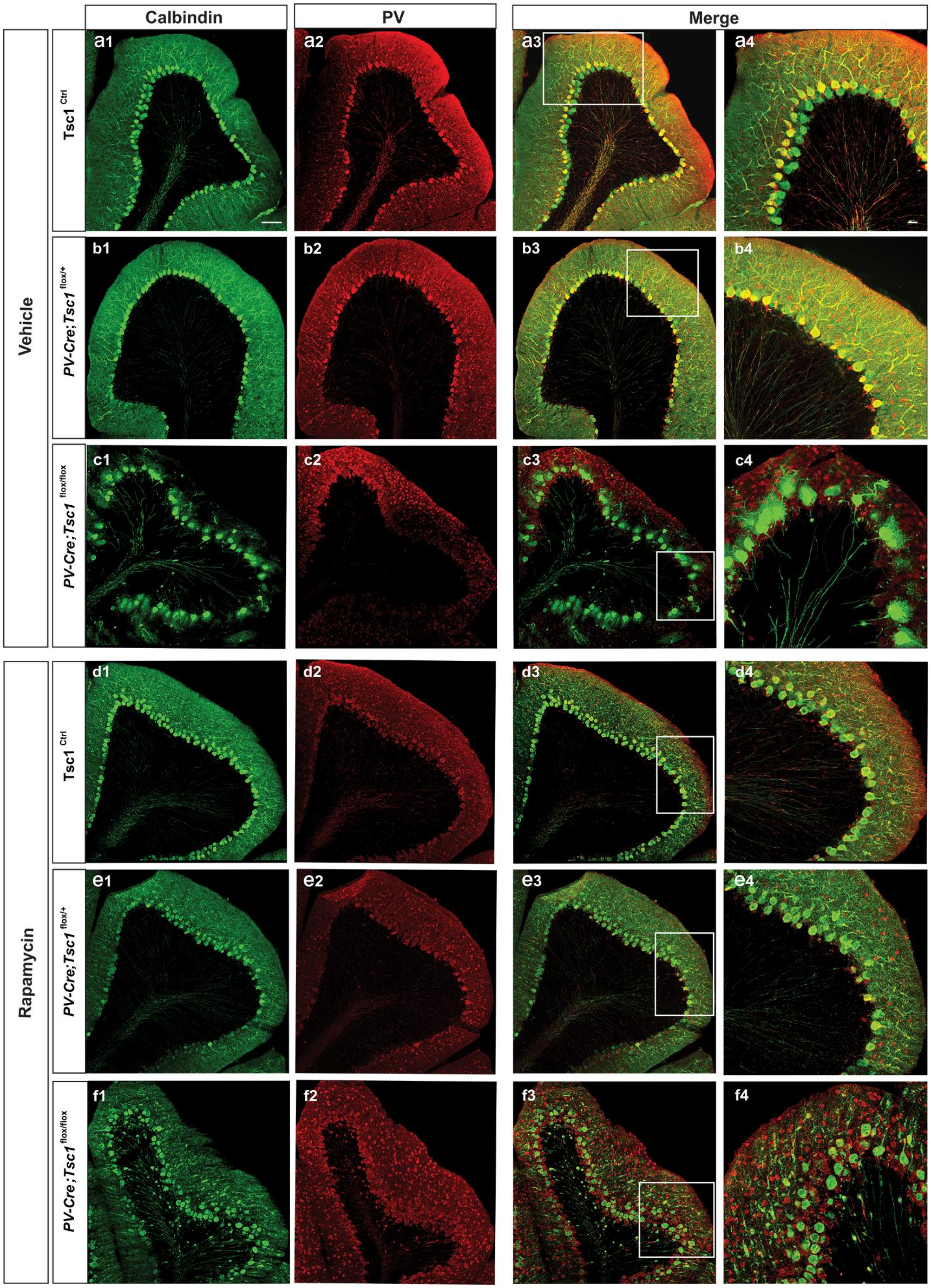
Calbindin
PV
Merge
Vehicle
Rapamycin
Tsc1 Ctrl
PV-Cre;Tsc1 flox/+
PV-Cre;Tsc1 flox/flox
Tsc1 Ctrl
PV-Cre;Tsc1 flox/+
PV-Cre;Tsc1 flox/flox
a1
a2
a3
a4
b1
b2
b3
b4
c1
c2
c3
c4
d1
d2
d3
d4
e1
e2
e3
e4
f1
f2
f3
f4

Supplementary Figure 11. Short term Rapamycin treatment does not rescue cerebellar abnormalities in adult homozygous mutant mice. Coronal sections of cerebellar cortex immunostained for Calbindin (green) and PV (red) in Vehicle treated *Tsc1*^{Ctrl} mice (**a1-a4**), *PV-Cre;Tsc1*$^{flox/+}$ mice (**b1-b4**) , *PV-Cre*;*Tsc1*$^{flox/flox}$ mice (**c1-c4**) and Rapamycin treated *Tsc1*^{Ctrl} mice (**d1-d4**), *PV-Cre*;*Tsc1*$^{flox/+}$ mice (**e1-e4**), *PV-Cre*;*Tsc1*$^{flox/flox}$ mice (**f1-f4**). Note that we did not observe any obvious abnormality in *PV-Cre*;*Tsc1*$^{flox/+}$ mice, while the *PV-Cre*;*Tsc1*$^{flox/flox}$ mice were significantly affected and the rescue with rapamycin treatment was only partial (**f4**). Scale bars: **a1-f3**, 10 µm; and **a4-f4**, 5 µm.
