# Supplementary material for: Sensitive period for rescuing parvalbumin interneurons connectivity and social behavior deficits caused by TSC1 loss
Source: Nat Commun. 2021 Jun 16;12:3653. doi: 10.1038/s41467-021-23939-7 (PMC8209106; doi:10.1038/s41467-021-23939-7)
Supplement: Supplementary file 3 — Reporting Summary [file 41467_2021_23939_MOESM3_ESM.pdf]

nature research

Corresponding author(s): Graziella Di Cristo

Last updated by author(s): 2021/02/25

Reporting Summary

Nature Research wishes to improve the reproducibility of the work that we publish. This form provides structure for consistency and transparency in reporting. For further information on Nature Research policies, see our Editorial Policies and the Editorial Policy Checklist.

Statistics

For all statistical analyses, confirm that the following items are present in the figure legend, table legend, main text, or Methods section.

n/a	Confirmed	
☐	☒	The exact sample size (*n*) for each experimental group/condition, given as a discrete number and unit of measurement
☐	☒	A statement on whether measurements were taken from distinct samples or whether the same sample was measured repeatedly
☐	☒	The statistical test(s) used AND whether they are one- or two-sided<br>*Only common tests should be described solely by name; describe more complex techniques in the Methods section.*
☒	☐	A description of all covariates tested
☐	☒	A description of any assumptions or corrections, such as tests of normality and adjustment for multiple comparisons
☐	☒	A full description of the statistical parameters including central tendency (e.g. means) or other basic estimates (e.g. regression coefficient) AND variation (e.g. standard deviation) or associated estimates of uncertainty (e.g. confidence intervals)
☐	☒	For null hypothesis testing, the test statistic (e.g. *F*, *t*, *r*) with confidence intervals, effect sizes, degrees of freedom and *P* value noted<br>*Give P values as exact values whenever suitable.*
☒	☐	For Bayesian analysis, information on the choice of priors and Markov chain Monte Carlo settings
☒	☐	For hierarchical and complex designs, identification of the appropriate level for tests and full reporting of outcomes
☒	☐	Estimates of effect sizes (e.g. Cohen's *d*, Pearson's *r*), indicating how they were calculated

Our web collection on statistics for biologists contains articles on many of the points above.

Software and code

Policy information about availability of computer code

Data collection	Analysis of mouse behavior in open field and elevated plus maze: SMART video tracking system (v3.0, Harvard Apparatus). EM data acquisition: Philips CM100 electron microscope, equipped with an 8 MB digital camera (AMT XR80). Confocal imaging: LAS X (Leica Application Suite X) version 8. PV cell arborisation analysis: Neurolucida (MBF Software version 9).
Data analysis	Statistical analysis: Prism 7.0 (GraphPad Software). Analysis of immunolabelled synaptic markers and western blot bands: ImageJ/Fiji: ImageJ1.53c (Open source software). Polygon tool: Leica Application Suite X, version 8.

For manuscripts utilizing custom algorithms or software that are central to the research but not yet described in published literature, software must be made available to editors and reviewers. We strongly encourage code deposition in a community repository (e.g. GitHub). See the Nature Research guidelines for submitting code & software for further information.

Data

Policy information about availability of data

All manuscripts must include a data availability statement. This statement should provide the following information, where applicable:

- Accession codes, unique identifiers, or web links for publicly available datasets
- A list of figures that have associated raw data
- A description of any restrictions on data availability

Detailed statistic and all data generated and analysed in the article are available from the corresponding author on request.

Field-specific reporting

Please select the one below that is the best fit for your research. If you are not sure, read the appropriate sections before making your selection.

- [x] Life sciences
- [ ] Behavioural & social sciences
- [ ] Ecological, evolutionary & environmental sciences

For a reference copy of the document with all sections, see nature.com/documents/nr-reporting-summary-flat.pdf

Life sciences study design

All studies must disclose on these points even when the disclosure is negative.

Sample size	Power analysis was used to determine appropriate sample size.
Data exclusions	In the analysis of social behavior, mice that refused to explore or that consistently remained on one side of the 3-chambers box where excluded from the analysis. In average one out of 15 mice refused to explore or move in this test and this behavior was independent of the genotype or treatment.
Replication	All attempts at replication were successful. All N of samples indicated in the figures are biologically independent.
Randomization	Mice or organotypic cultures were randomly assigned to experimental groups.
Blinding	For behavioral experiments and western blot analysis , experimentalists were blinded to group allocation during data collection and analysis. For confocal imaging experiments in vivo and in organotypic cultures, blinding of experimentalists during data collection was not possible because the same person prepared the tissue and imaged it. However, care was taken to maintain imaging parameters consistent across all experimental groups. In addition, all data analysis was performed by lab members that were blinded to genotype and/or treatment.

Reporting for specific materials, systems and methods

We require information from authors about some types of materials, experimental systems and methods used in many studies. Here, indicate whether each material, system or method listed is relevant to your study. If you are not sure if a list item applies to your research, read the appropriate section before selecting a response.

Materials & experimental systems

n/a	Involved in the study	
[ ]	[x]	Antibodies
[x]	[ ]	Eukaryotic cell lines
[x]	[ ]	Palaeontology and archaeology
[ ]	[x]	Animals and other organisms
[x]	[ ]	Human research participants
[x]	[ ]	Clinical data
[x]	[ ]	Dual use research of concern

Methods

n/a	Involved in the study	
[x]	[ ]	ChIP-seq
[x]	[ ]	Flow cytometry
[x]	[ ]	MRI-based neuroimaging

Antibodies

Antibodies used	Primary antibody for Immunolabeling: Rabbit anti-phospho-S6 (Cell Signaling, Cat# 5364), mouse anti-NeuN (Millipore, Cat# MAB377), chicken anti-NeuN (Millipore, Cat# ABN91), mouse anti-PV (Swant, Cat# 235), rabbit anti-PV (Swant, Cat# PV27), guinea pig anti-PV (Synaptic Systems, Cat# 195004), mouse anti-gephyrin (Synaptic Systems, Cat# 147021), rabbit anti-VGAT (Synaptic System, Cat #131003), chicken anti-GFP (1:1000, Abcam, Cat#13970), mouse anti-Calbindin (Abcam, cat#9481).<br><br>Primary antibody for western blot: rabbit anti-LC3B (Novus, Cat #NB100-2220), rabbit anti-p62 (Proteintech, Cat#18420-1-AP), anti-pAMPK (T172, Cell Signaling, cat#2535), anti-AMPK (Cell Signaling, cat#2532), rabbit anti-ULK1 (D8H5; Cell Signaling, cat#8054), anti-pULK1 (Ser555, D1H4; Cell Signaling, cat#5869), mouse anti-GAPDH (1:5000, ThermoFisher, Cat#MA5-15738).<br><br>Secondary antibodies: Goat anti-Mouse 488 (Cell Signaling, cat#4408S), Goat anti-Mouse 555 (Cell Signaling, cat#4409S). Goat anti-Rabbit 488 (Life Technologies , cat#A11008), Goat anti-Mouse 594 (Life Technologies , cat#A11020), Goat anti-Guinea Pig 647 (Life Technologies , cat# A21125), Goat anti-Rabbit 633 (Life Technologies , cat#A21072), Goat anti-Rabbit 555 (Life Technologies , cat#A21430), Goat anti-chicken 488 (Abcam, cat#Ab150169).
Validation	Information can be found in the antibody registrym (antibodyregistry.org), using RRID numbers. When RRDI is not available, we refer to published work.

Rabbit anti-phospho-S6 (Cell Signaling, Cat# 5364), RRID:AB_10694233
mouse anti-NeuN (Millipore, Cat# MAB377), RRID:AB_2298772
chicken anti-NeuN (Millipore, Cat# ABN91), RRID:AB_11205760
mouse anti-PV (Swant, Cat# 235), RRID:AB_10000343,
rabbit anti-PV (Swant, Cat# PV27), RRID:AB_2631173
guinea pig anti-PV (Synaptic Systems, Cat# 195004), RRID:AB_2156476
mouse anti-gephyrin (Synaptic Systems, Cat# 147021), RRID:AB_2232546
rabbit anti-VGAT (Synaptic System, Cat #131003), RRID:AB_887869
chicken anti-GFP (Abcam, Cat#13970), RRID:AB_300798
rabbit anti-LC3B (Novus, Cat #NB100-2220), RRID: AB_10003146
rabbit anti-p62 (Proteintech, Cat#18420-1-AP), RRID:AB_10694431
rabbit anti-pAMPK (T172, Cell Signaling, cat#2535), PMID: 24599401
anti-AMPK (Cell Signaling, cat#2532), PMID: 24599401
rabbit anti-ULK1 (D8H5; Cell Signaling, cat#8054), RRID:AB_11178668
anti-pULK1 (Ser555, D1H4; Cell Signaling, cat#5869), PMID: 24599401
mouse anti-GAPDH (1:5000, ThermoFisher, Cat#MA5-15738), RRID:AB_10977387,
mouse anti-Calbindin (Abcam, cat#9481), RRID:AB_2811302.

All secondary antibodies have been validated by the respective manufacturers.

Animals and other organisms

Policy information about studies involving animals; ARRIVE guidelines recommended for reporting animal research

Laboratory animals	Tsc1 floxed mice with loxP sites flanking exons 17 & 18 of Tsc1 gene (Tsc1flox/flox) were purchased from Jackson Laboratories (Cat# 005680). Two separate driver mouse lines expressing Cre recombinase, (1) Tg(Nkx2.1-Cre), (Jackson Laboratories, Cat# 008661) and (2) PV-Cre (Jackson Laboratories, Cat# 008069) were crossed to the Tsc1 floxed mice and the respective progenies were backcrossed to generate the heterozygous, homozygous and control genotypes within the same litter. To control for the pattern of expression of Cre, we introduced the RCE allele using Gt(ROSA)26Sortm1.1(CAG-EGFP)Fsh/J mice (Jackson laboratories). All mice were housed under standard pathogen-free conditions in a 12h light/dark cycle, 21 C and 40% humidity, and with ad libitum access to sterilized laboratory chow diet. Post-weaning, two to five mice were housed in a single cage. We used mice of both sexes across the study. Specific ages of mice are reported in the manuscript for each experiment.
Wild animals	The study did not involve wild animals
Field-collected samples	The study did not involve field collected samples.
Ethics oversight	Animals were treated in accordance with Canadian Council for Animal Care and protocols were approved by the Animal Care Committee of CHU Ste-Justine Research Center.

Note that full information on the approval of the study protocol must also be provided in the manuscript.
